# Cocaine-Induced Reinstatement of a Conditioned Place Preference in Developing Rats: Involvement of the D2 Receptor

**DOI:** 10.3390/brainsci2040573

**Published:** 2012-10-31

**Authors:** Kimberly A. Badanich, Cheryl L. Kirstein

**Affiliations:** 1 Department of Psychology, College of Arts and Sciences, University of South Florida Sarasota-Manatee, 8350 N. Tamiami Trail, Sarasota, FL 34243, USA; 2 Department of Psychology, Cognitive and Neural Sciences, University of South Florida, 4202 E. Fowler Avenue, Tampa, FL 33620, USA; Email: kirstein@usf.edu; 3 Department of Molecular Pharmacology and Physiology, University of South Florida College of Medicine, Tampa, FL 33612, USA

**Keywords:** ontogeny, adolescent rat, nucleus accumbens, dopamine, reinstatement

## Abstract

Reinstatement of conditioned place preferences have been used to investigate physiological mechanisms mediating drug-seeking behavior in adolescent and adult rodents; however, it is still unclear how psychostimulant exposure during adolescence affects neuron communication and whether these changes would elicit enhanced drug-seeking behavior later in adulthood. The present study determined whether the effects of intra-ventral tegmental area (VTA) or intra-nucleus accumbens septi (NAcc) dopamine (DA) D2 receptor antagonist infusions would block (or potentiate) cocaine-induced reinstatement of conditioned place preferences. Adolescent rats (postnatal day (PND 28–39)) were trained to express a cocaine place preference. The involvement of D2 receptors on cocaine-induced reinstatement was determined by intra-VTA or intra-NAcc infusion of the DA D2 receptor antagonist sulpiride (100 μM) during a cocaine-primed reinstatement test (10 mg/kg cocaine, *i.p*.). Infusion of sulpiride into the VTA but not the NAcc blocked reinstatement of conditioned place preference. These data suggest intrinsic compensatory mechanisms in the mesolimbic DA pathway mediate responsivity to cocaine-induced reinstatement of a conditioned place preference during development.

## 1. Introduction

Place conditioning and cocaine-induced reinstatement paradigms have provided a measure of drug reward by assessing an animal’s ability to associate drug-induced effects with environmental cues. Cocaine place preferences were effectively established for adolescent and adult rodents at standard cocaine doses [1,2,3,4]. Balda and colleagues [4] were the first to demonstrate adolescent mice were able to express cocaine-induced reinstatement of cocaine place conditioning while a more recent report suggested age effects for cocaine-induced reinstatement of a conditioned place preference were dose dependent [5]. However, when lower doses were tested, early adolescents demonstrated place preferences for lower doses of cocaine than late adolescent and adult rats indicating that early adolescent rats were more responsive to the rewarding properties of a low dose of cocaine than older rats [3]. Long-term effects of early cocaine exposure on reinstatement are unknown and the propensity for cocaine exposure to alter sensitivity to drug relapse during adulthood has yet to be determined. 

During development, major changes occur in dopamine (DA) receptor/transporter density [6,7,8,9,10], sensitivity of DA autoreceptors [11] rate of DA synthesis/degradation [12,13,14,15] and basal DA levels ([3]; but see [16]). Recently *in vivo* microdialysis has shown psychostimulants increased DA in the nucleus accumbens septi (NAcc) of preadolescent [17] and adolescent rodents [3,16,18,19]. Interestingly, Brenhouse and colleagues [20] demonstrated the density of glutamatergic projection cells from the prefrontal (PFC) to the NAcc core progressively increased across age with adults having greater cortical innervations of the NAcc than younger rats. Given that the ventral tegmental area (VTA), prefrontal cortex (PFC) and NAcc core mediate cocaine-induced reinstatement [21,22,23,24,25,26] and it is these same brain regions that undergo developmental transitions during adolescence [20], investigating the involvement of these brain regions in the long-term effects of early cocaine exposure on reinstatement was warranted. 

Much research has been conducted on the involvement of DA receptors and transporters in cocaine-induced reinstatement and DA levels for adult rodents [27,28]. Systemically administered low dose D1 agonists, D3 agonists or local administration of D1 agonists into the NAcc shell reinstated cocaine-seeking whereas systemically administered high dose D1 agonists or D1, D2, D3 antagonists attenuated cocaine seeking behavior [29,30]. The present study examined the function of VTA and NAcc DA autoreceptors in adult rats previously exposed to cocaine during adolescence. The effects of intra-VTA or intra-NAcc DA D2 antagonist infusions (sulpiride) on cocaine-induced reinstatement of cocaine place conditioning in the developing rat were examined.

## 2. Methods and Materials

### 2.1. Subjects

Male and female breeders were obtained from Harlan Laboratories for the purpose of producing adolescent rats. Breeding pairs were established and all rats used in the present study were bred from these pairs at the University of South Florida. In house breeding was chosen instead of ordering young rats directly from a vendor to control for any possible stress effects on the developing animal during shipment to our facility. From these breeding pairs, a total of sixty-three male Sprague-Dawley pups were used as experimental subjects in the present study. The day of birth was designated as postnatal day (PND) 0 and litters were sexed and culled to 10 pups per litter on PND 1. The number of males per litter ranged from 5 to 8 males per litter of 10 pups. Pups remained housed with their respective dams in a temperature and humidity-controlled vivarium on a 12:12-h light/dark cycle (lights on from 7:00 a.m. to 19:00 p.m.). On PND 21, pups were weaned and housed in groups of two or three with same-sex littermates. Adolescence in rodents is encompassed within approximately PND 28 to PND 46 and is marked by several developmental events including the onset of puberty and changes in neuroendocrine systems in addition to increased socialization and exploratory behaviors [31,32]. Therefore, to investigate the effects of cocaine on adolescent behavior, rats in the present study began training on PND 28 (adolescence), received drug treatment during adolescence (PND 30–39) and behavioral tests through to adulthood (PND 40–75). Body weight for adolescent rats bred in our vivarium averaged 91 g on the first day of the experimental protocol. To eliminate the potential confound of litter effects, no more than one pup per litter was used for any given condition and remaining pups were used for other ongoing laboratory experiments. In all respects, the maintenance and treatment of rats were within the guidelines for animal care as approved by the University of South Florida’s Institutional Animal Care and Use Committee and according to the National Institutes of Health. 

### 2.2. Apparatus

The place conditioning apparatus was a single runway comprised of clear acrylic. The apparatus was one large chamber that could be separated into two smaller environments by inserting a removable solid divider. Each environment (21 × 24.5 × 20.5 cm) was comprised of different removable visual and tactile cues. One environment had black and white horizontal striped (1 inch thick) walls with a grey sandpaper floor while the alternate environment had black and white vertical striped walls (1 inch thick) with a wire-mesh floor. The solid divider separating the two environments was only present during conditioning and was removed during baseline and place preference testing. A 2-environment apparatus, rather than a 3-environment, was used to eliminate age-related differences in novelty-induced exploration [33] that may likely be induced by a less familiar central choice environment, which is typically incorporated in the 3-environment conditioned place preference (CPP) paradigm. The place conditioning apparatus was used during place conditioning acquisition, extinction and cocaine-induced reinstatement tests. Prior to cocaine treatment, baseline preferences for the two sides of the apparatus were slightly biased towards the vertical striped/wire environment (60% of rats tested preferred the vertical/wire environment).

### 2.3. Procedure

Cocaine place conditioning and local microinjections were used to investigate the underlying physiological mechanisms inducing cocaine-induced reinstatement of drug seeking behaviors. Experiment One consisted of four phases including (1) place conditioning (2) extinction, (3) surgery, (4) local infusion and reinstatement test (see Table 1 for details). For all rats, the experiment began on PND 28 and each rat was investigated at all four phases. Procedures for each of the four phases are described below.

**Table 1:** Experimental design.

Age (Postnatal day, PND)	30–39	40	65	70
Phase	Conditioned place preference (CPP)	Extinction	Surgery	Reinstatement
	saline		NAcc	saline
	saline		NAcc	sulpiride
	cocaine		NAcc	saline
	cocaine		NAcc	sulpiride
	saline		VTA	saline
	saline		VTA	sulpiride
	cocaine		VTA	saline
	cocaine		VTA	sulpiride

*Phase 1: Acquisition and Expression of Cocaine Place Conditioning.* On days one and two (PND 28 and 29), rats were gently handled for three minutes so that rats became used to the experimenter. Place conditioning was conducted on days three through twelve (PND 30–39) and consisted of three phases: baseline (day three), drug conditioning (days four-eleven) and a CPP expression test (day twelve). On day three (PND 30), a biased design was used to determine baseline environment preferences. Time (s) spent in each environment and distance traveled (cm) were recorded by tracking where the rat was located in the apparatus (*i.e.*, paired *vs.* unpaired environment) and calculating total time spent in each environment (Ethovision video tracking system, Noldus, Netherlands). Location of the rat was determined when the center of the rat’s body crossed over the environment parameter and specifically aided in designating location of rats straddling both environments. The center of the rat was defined as the middle of the rat’s body excluding the tail. The environment in which each animal spent the least amount of time at baseline was designated as the cocaine-paired environment for future trials. Starting on the morning of day four (PND 31), rats were injected with 20 mg/kg cocaine intraperitoneally and confined to the cocaine-paired environment for fifteen minutes. The 20 mg/kg cocaine dose has been previously shown to effectively establish a CPP in adolescent rats [3]. On the following day, once the effects of cocaine had dissipated, rats were injected with saline and confined to the alternate cued environment for fifteen minutes. Control rats received saline injections in both environments. Place conditioning occurred once a day over eight consecutive days (PND 31–38) for a total of four cocaine and four saline exposures. The apparatus was cleaned prior to each trial to remove odors. On day twelve (PND 39), the conditioned effects of cocaine were tested by administration of a CPP expression test. Rats were administered a saline injection and tested drug-free in the same manner as at baseline (day three). A saline injection was given during the CPP expression test to control for any associations that the injection may have acquired during CPP acquisition. A total of 18 rats were excluded from the analyses because they did not condition (*i.e.*, no CPP expression).

*Phase 2: Extinction.* In order to extinguish the association between cocaine and the environmental cues, rats were repeatedly tested in the same manner as during the CPP expression test (day thirteen; PND 40). Each rat was tested daily for extinction until the subject spent relatively equal amounts of time in the paired and unpaired environments. Extinction was defined when rats no longer expressed a preference for either environment (*i.e.*, 44%–55% of entire trial spent in one environment). All rats eventually extinguished the association between the paired environment and cocaine effects and proceeded to phase 3 of the experiment.

**Figure 1:**
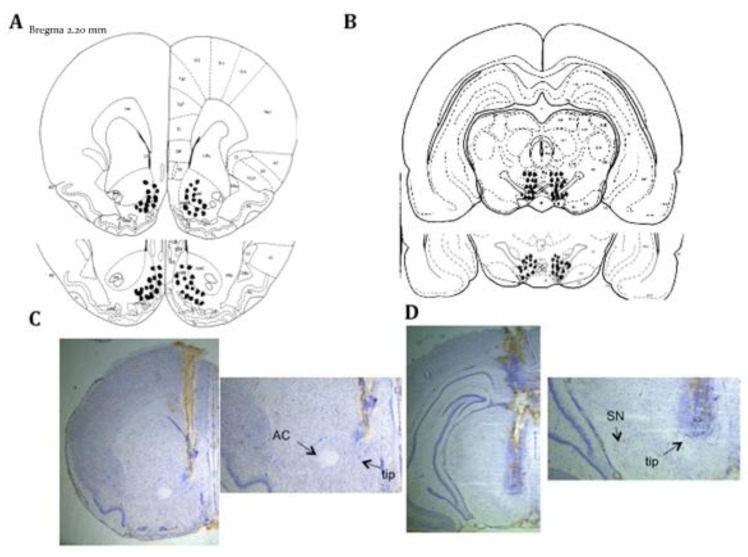
Dropsites for ventral tegmental area (VTA) and nucleus accumbens septi (NAcc) Injector Cannula. Placement of injector cannula into the NAcc and VTA. Dots represent the deepest portion of the injector cannula tip. Some dots represent more than one dropsite. (**A**) NAcc injector cannula placement in rats tested for reinstatement; *n* = 31. Plates = +2.20, +1.70 mm. (**B**) VTA injector cannula placement in rats tested for reinstatement; *n* = 32. Plates = −3.40 and −3.60 mm. Example photographs of injector placement are shown in the (**C**) NAcc and (**D**) VTA. Tip = bottom of injector tip, SN = substantia nigra, AC = anterior commissure.

*Phase 3: Surgical Procedures.* Our lab has consistently used weight based coordinates to aim microdialysis guide cannula at the NAcc of adolescent rats due to the fact that adolescents rapidly grow in size and gain a substantial amount of weight during development that ultimately affects accurate placement of microdialysis probes in target brain regions [3,18,34,35,36,37] and the VTA [38]. Since our previous microdialysis experiments have also included adult comparisons, we have observed using these weight based coordinates for adult rats provide much greater accuracy in placing cannula in both the NAcc and VTA for adult rats as well. Therefore, weight based coordinates were used for adult rats in the present experiments. On PND 65, rats were anesthetized with ketamine/xylazine (75 mg/kg ketamine, 5 mg/kg xylazine, *i.p.*), an incision was made over the skull and the rat was mounted on a stereotaxic instrument for surgery (myNeurolab, St Louis, MO). Specifically for NAcc cannula placements, the head was leveled and holes for bilateral guide cannula were drilled in the skull at a site above the NAcc (Plastics One, 10.5 mm guides, 22 gauge, 1.5 mm separation). Two holes for skull screws were drilled in the skull adjacent to the guide cannula as well. A guide cannula equipped with a dummy cannula (for CMA 11 probes; outer diameter 0.6 mm) was lowered into the brain to a site just above the anterior NAcc (mean weight based coordinate measured from bregma: anterior (+2.3); lateral (+0.7); ventral (−5.6)) and affixed to the skull with cranioplast. The dorsal-ventral coordinate for NAcc coordinates were measured from the skull surface and allowed room for later insertion of the 2mm microinjection tip. During histology (see below), NAcc guide cannula dropsites were compared to Paxinos and Watson’s rat brain atlas [39] for verification of proper placement in the NAcc. For VTA cannula placements, weight-based coordinates were generated according to Pellegrino, Pellegrino and Cushman [40]. Specifically, rats were placed in the stereotax so that the interaural line was 5.0 mm below the upper incisor bar. Holes for bilateral guide cannula were drilled in the skull at a site above the VTA (Plastics One, 10.5 mm guides, 22 gauge, 2.0 mm separation). Two holes for skull screws were drilled in the skull adjacent to the guide cannula as well. A guide cannula equipped with a dummy cannula (for CMA 11 probes; outer diameter 0.6 mm) was lowered into the brain to a site just above the VTA [from bregma: posterior (−3.5); lateral (+1.0); ventral (−6.0)] and affixed to the skull with cranioplast. The dorsal-ventral coordinate for NAcc coordinates were measured from the skull surface and allowed room for later insertion of the 2 mm microinjection tip. During histology (see below), VTA guide cannula dropsites were compared to Pellegrino, Pellegrino and Cushman’s rat brain atlas [40] for verification of proper placement in the VTA. Note that NAcc and VTA coordinates listed above are averages given that weight based coordinates were used [34,38]. It should be noted that the anterior NAcc was specifically targeted given its suggested role in incentive salience [41]. Furthermore, we specifically hit the PN & PBP nuclei of the VTA because these nuclei send projections to the NAcc. These two nuclei are located primarily in the anterior portion of the VTA. Guide cannulae placements for the VTA and NAcc are illustrated with example photographs in Figure 1. Following surgery, rats were singly housed in the home cage and allowed at least 4 days for recovery.

*Phase 4: Intra-VTA or Intra-NAcc Infusions of the D2 Antagonist Sulpiride on Cocaine-Induced Reinstatement of Cocaine Place Conditioning.* On reinstatement test day (PND 70), the dummy cannula was removed and an injection cannula was inserted into the brain region of interest (VTA, NAcc). The injection cannula (Plastics One, 28 gauge, 2 mm projection tip, 2.0 mm separation for VTA and 1.5 mm separation for NAcc) was connected via dialysis tubing to a syringe pump set to a flow rate of 0.5 μL/min. The syringe was filled with either saline alone or saline + 100 μM sulpiride [42]. Each perfusate was injected into the appropriate brain region for 1 min, followed by turning the syringe pump off. The injector cannula remained inserted for an additional 2 min to facilitate diffusion of the perfusate into the brain tissue and to prevent the perfusate from drawing up into the guide cannula shaft. Following local perfusions, the injector cannula was removed and replaced with the dummy cannula. These procedures have been used before to successfully infuse drugs into NAcc [43,44]. Rats were placed back into their homecage for 30 min to allow infusions to have a maximal effect on accumbal DA [42] and to allow time before a subsequent cocaine-induced reinstatement test. 

Cocaine-induced reinstatement of cocaine place preferences were investigated by reintroducing rats to cocaine associated cues in the conditioning apparatus immediately following a systemic cocaine prime. Following the local infusion of sulpiride and the 30 min waiting period, rats were tested for cocaine prime-induced reinstatement by administering a priming injection of cocaine (10 mg/kg, *i.p.*) and reintroducing rats to the cocaine-paired cues by placing them inside the place conditioning apparatus. Once inside the place conditioning environment, all rats were tested in the same manner as during the CPP expression test (phase 1, day twelve). 

### 2.4. Histological Verification

Immediately following the reinstatement test, rats were euthanatized and brains were removed to histologically verify that guide cannula were accurately placed in the VTA or NAcc. Histological verification was conducted by removing and freezing the brain and subsequently cutting the frozen tissue into 40 μm sections using a cryostat. Brain sections were mounted on slides, stained with cresyl violet and examined with brightfield microscopy. Accurate placement of guide cannula tracts was identified by comparing each brain section to a rat brain atlas [39,40]. 

### 2.5. Design and Analyses

**Figure 2:**
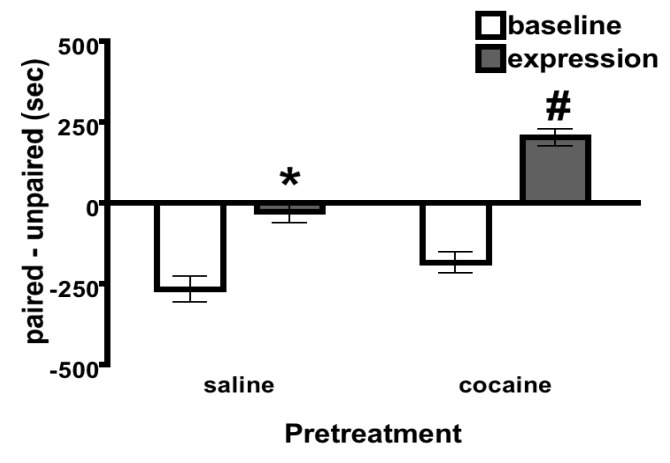
Place Conditioning. Cocaine place conditioning during adolescence. Cocaine treated adolescent rats showed a place preference. * differs from baseline. ^#^ differs from baseline and saline. Bars denote means and SEM. Sample sizes = 31 or 32 rats per group.

**Figure 3:**
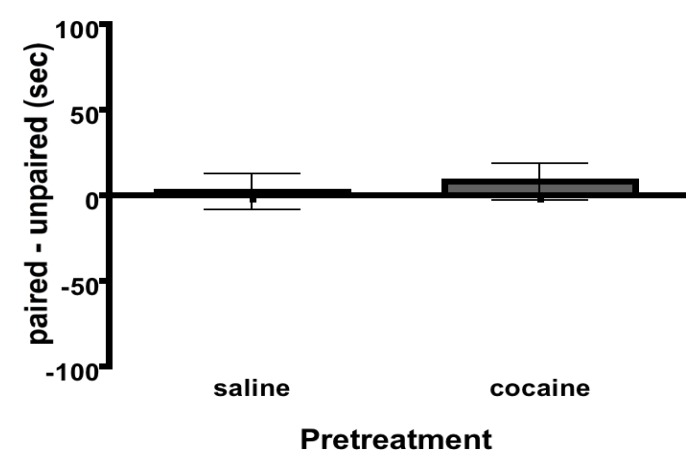
Extinction. All rats extinguish. There were no effects of Pretreatment on extinction. Same rats from Figure 2. Bars denote means and SEM.

**Figure 4:**
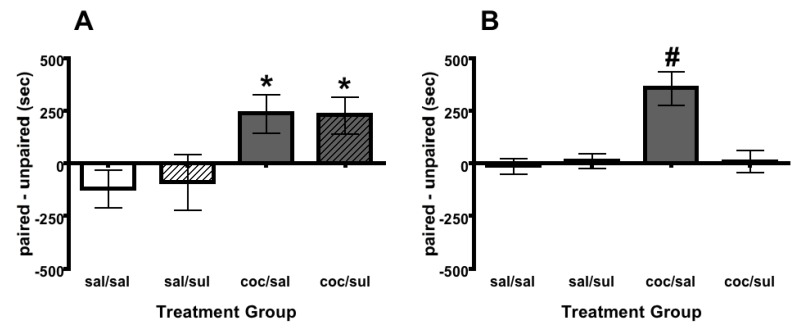
Effects of Intra-NAcc or Intra-VTA Sulpiride on Reinstatement. (**A**) Adult rats with a history of early cocaine exposure showed cocaine induced reinstatement of cocaine place conditioning (grey bars; indicated by *). There was no effect of intra-NAcc sulpiride on cocaine-induced reinstatement of cocaine place conditioning (grey striped bars; indicated by *). Sample sizes = 8 sal/sal; 7 sal/sul; 8 coc/sal; 8 coc/sul. (**B**) Adult rats with a history of early cocaine exposure showed cocaine induced reinstatement of cocaine place conditioning (grey bars; indicated by ^#^). Intra-VTA sulpiride blocked cocaine-induced reinstatement of cocaine place conditioning (grey striped bars). Sample sizes = 8 sal/sal; 8 sal/sul; 8 coc/sal; 8 coc/sul. NAcc = nucleus accumbens, VTA = ventral tegmental area, sal = saline, sul = sulpiride and coc = cocaine. All bars denote means and SEM.

It was the aim of the present study to determine whether cocaine-induced reinstatement of cocaine place conditioning was mediated by D2 receptors in the VTA and NAcc. Pretreatment (saline *vs.* cocaine) and Group (sal/sal; sal/sul; coc/sal; coc/sul) were between subjects variables and Session (baseline *vs.* expression) was the repeated measure. The dependent measure for place conditioning, extinction and reinstatement was time spent in the paired environment expressed as a difference score (second in paired environment − second in unpaired environment). A two-way analyses of variance (ANOVA) was used to analyze place conditioning scores (mixed model: Session (baseline *vs.* expression) × Pretreatment (saline *vs.* cocaine)). An unpaired *t*-test of Pretreatment effects was used to evaluate extinction scores. One way-between subjects ANOVA of Group were used to analyze reinstatement scores in VTA and NAcc infused rats. Simple effects (for Figure 2) and Fisher’s least significant difference (LSD) *post hoc* analyses (for Figure 2, Figure 3 and Figure 4) were used to isolate Session, Pretreatment, and Group effects. All statistical analyses were determined significant at the 0.05 alpha level. 

## 3. Results

### 3.1. Cocaine Place Conditioning in Adolescent Rats

All rats were exposed to conditioned place preference training during adolescence (PND 30–38). Time spent (s) and distance traveled (cm) in the paired environment on test day (PND 39) were recorded as the conditioned place preference and locomotor activity scores. There was a significant Pretreatment × Session interaction for time (*F*(1, 60) = 5.48, *P* < 0.05) that revealed adolescent rats showed a clear preference for the cocaine-paired environment (Figure 2). Simple effects of Session and Fisher’s LSD *post hoc* analyses revealed time spent in the paired environment was greater during expression than at baseline regardless of Pretreatment group (*P* < 0.05). However, simple effects of Pretreatment and Fisher’s LSD *post hoc* analyses revealed rats pretreated with 20 mg/kg cocaine *i.p.* spent more time in the paired environment than saline-pretreated rats (*P* < 0.05; indicated by ^#^). Therefore, cocaine-treated adolescent rats showed a place preference. There was no interaction between Pretreatment and Session for distance traveled (cm) in the paired environment during the place conditioning expression test (*F*(1, 60) = 2.21, *P* > 0.05; data not shown). There was a significant Main Effect of Pretreatment (*F*(1, 60) = 9.15, *P* < 0.05) and a significant Main Effect of Session (*F*(1, 60) = 23.79, *P* < 0.05) that indicated a moderate ability of cocaine to enhance locomotor activity during the place preference expression test (cocaine = 2012 ± 56 cm; saline = 1667 ± 67 cm).

### 3.2. Extinction

Immediately following place preference testing during adolescence (PND 40), the conditioned effects of cocaine were extinguished for all rats. Extinction trials continued for each rat until they spent equal amounts of time in the paired and unpaired environments. The group average for the number of days it took rats to extinguish the association between cocaine and environmental cues was 5 ± 0.68 days. There were no effects of Pretreatment on time (Figure 3; *t*(60) = 0.37, *P* > 0.05) or distance traveled (*t*(60) = 0.03, *P* > 0.05; data not shown) for the last extinction trial. 

### 3.3. Effects of Intra-NAcc or Intra-VTA Sulpiride on the Reinstatement of Cocaine Place Preferences in Developing Rats

Following extinction, rats were left undisturbed until they reached adulthood (PND 65). Rats received an intra-NAcc infusion (100 μM sulpiride or saline), a priming systemic injection (10 mg/kg cocaine or saline, *i.p.*) and a reinstatement test (PND 70). There was a significant effect of Treatment Group when tested for reinstatement later on in adulthood (*F*(3, 27) = 3.77, *P* < 0.05). Fisher’s LSD *post hoc* analyses revealed the cocaine treated groups (coc/sal and coc/sul) spent more time in the paired environment than the saline groups ((sal/sal and sal/sul); *P* < 0.05; indicated by *). These data illustrate that a priming dose of cocaine (10 mg/kg, *i.p.*) and an intra-NAcc infusion of saline reinstated a cocaine place preference as shown by increased time spent in the paired environment for the coc/sal group (Figure 4A; grey bars). Reinstatement of cocaine place preference was not altered by intra-NAcc injections of sulpiride as shown by a similar increase in time spent in the paired environment for the coc/sul group (Figure 4A; grey striped bars). 

A separate group of rats were used for intra-VTA infusions. Following extinction, rats were left undisturbed until they reached adulthood (PND 65). Rats received an intra-VTA infusion (100 μM sulpiride or saline), a priming systemic injection (10 mg/kg cocaine or saline, *i.p.*) and a reinstatement test (PND 70). There was a significant effect of Treatment Group when tested for reinstatement later in adulthood (Figure 4B; group: *F*(3, 28) = 10.52, *P* < 0.05). Fisher’s LSD *post hoc* analyses revealed the coc/sal treated group spent more time on the paired environment than sal/sal, sal/sul and coc/sul groups (*P* < 0.05, indicated by ^#^). These data illustrate a priming dose of cocaine (10 mg/kg, *i.p.*) and an intra-VTA infusion of saline reinstated a cocaine place preference as shown by increased time spent in the paired environment for the coc/sal group (Figure 4B; grey bars). Reinstatement of cocaine place preference was blocked by intra-VTA injections of sulpiride as shown by a lack of increased time spent in the paired environment for the coc/sul group (Figure 4B; grey striped bars). 

There was a significant Main Effect of Treatment Group for distance traveled in the paired environment during the reinstatement test for both the NAcc-infused (Table 2: *F*(3, 27) = 6.73, *P* < 0.05) and VTA-infused (Table 2: *F*(3, 28) = 6.60, *P* < 0.05) groups. Overall, systemic cocaine enhanced locomotor activity during the reinstatement test. However, unlike the CPP expression test data, intra-NAcc and intra-VTA infusions of sulpiride were unable to block cocaine-induced increases in locomotor activity during the reinstatement test. 

**Table 2:** Locomotor activity levels during reinstatement. Scores denote mean ± SEM. * differs from sal/sal.

	sal/sal	sal/sul	coc/sal	coc/sul
**NAcc**	1065 ± 143	852 ± 98	2473 ± 495 *	2103 ± 261 *
**VTA**	1079 ± 215	1449 ± 213	1939 ± 166 *	2139 ± 141 *

## 4. Discussion

Cocaine-induced reinstatement of a conditioned place preference has been shown in adolescent rodents [4,5] with adolescents exhibiting enhanced sensitivity at lower cocaine doses [5]. However, long-term effects of early cocaine exposure on reinstatement were unknown and the propensity for cocaine exposure to alter sensitivity to drug relapse during adulthood had yet to be determined. The present study revealed intra-VTA, but not intra-NAcc, infusions of the D2 antagonist sulpiride blocked cocaine-induced reinstatement of cocaine place preferences in adult rats that were previously exposed to cocaine during adolescence (PND 31–38). Sulpiride did not block cocaine-induced increases in locomotor activity and suggests the involvement of the D2 receptor in the expression of cocaine-primed reinstatement is not due to changes in locomotor activity. Given that intra-VTA infusions of a D2 antagonist blocked reinstatement, it can be suggested that D2 receptors in the VTA are one major factor mediating cocaine-seeking behaviors in adult rats previously exposed to cocaine during adolescence. 

It should be noted that saline treated control rats exhibited greater preference for the “paired chamber” during the CPP expression test. All rats, cocaine treated and control rats, were initially biased towards one environment during the baseline test indicating rats spent more time in one environment over the other. During conditioning, control rats were repeatedly exposed to the same null treatment (*i.e.*, saline injections) in both environments. As a result, control rats had difference scores near zero indicating they were spending equal amounts of time in each chamber. Given that control rats were treated in the same manner in both environments, it is only to be expected that rats would habituate to the apparatus and the initial bias or preference for one environment over another would diminish, leaving control rats to respond to each environment in the same manner. On the other hand, cocaine treated rats were repeatedly exposed to cocaine in each rat’s initially least preferred environment. It is likely that some habituation effects were also present in the cocaine treated rats. However, the fact that cocaine treated rats not only increased the amount of time they spent in the cocaine-paired environment (relative to baseline) but they also spent more time in this environment than in the alternative environment (relative to control rats) indicates cocaine treated rats had a valid preference for the cocaine paired environment. 

DA agonists/antagonists alter cue-induced reinstatement of cocaine seeking. Systemically administered low dose D1 agonists, D3 agonists or local administration of D1 agonists into the nucleus accumbens shell reinstate cocaine seeking whereas systemically administered high dose D1 agonists or D1, D2, D3 antagonists attenuate cocaine seeking behavior [29,30]. There is a paucity of data implicating the role of VTA D2 antagonists on cocaine place conditioning in the rat. D2 autoreceptors are highly expressed in the VTA and are primarily located on DA neurons [45,46]. Given that intra-VTA infusions of a D2 antagonist blocked reinstatement in rats with a history of early cocaine exposure but intra-NAcc infusions were without effect suggests D2 receptors in these two brain regions play different roles in cocaine relapse. Normal functioning of NAcc D2 receptors may be disrupted by early cocaine exposure and may be unresponsive to locally infused D2 antagonists. However, VTA D2 antagonists may still be able to function in a way that allows D2 antagonists to block cocaine relapse. There are glutamatergic innervations and GABAergic interneurons in both the VTA and NAcc [47,48,49] and these neurotransmitters systems may interact with DA pathways to play a role in cocaine relapse for rodents previously exposed to cocaine early in life. 

Since only VTA D2 receptors were found to mediate cocaine-induced reinstatement of cocaine place conditioning in developing rats, it is likely that the VTA and NAcc differ in D2 receptor density, functionality or intracellular signaling mechanisms following exposure to cocaine during adolescence. The role of VTA D2 receptors in mediating cocaine-induced reinstatement of cocaine place conditioning for developing rats is likely potentiated by altered levels of D2 receptors or Gi-alpha proteins in the VTA that couple to D2 receptors and inhibit adenylyl cyclase. Atypical functionality or density of D2 receptors may be even more pronounced in the VTA than in the NAcc of adult rats with a history of early cocaine exposure given that the present data showed intra-VTA but not intra-NAcc D2 antagonist infusions blocked cocaine-induced reinstatement. These data suggest D2 receptors specifically in the VTA of rats with a history of early cocaine exposure may mediate cocaine sensitivity and possibly cocaine relapse later on in adulthood.

In the present study, blockade of D2 receptors in the VTA likely altered mesocortical as well as mesolimbic DA neuron function. D2 receptors in the VTA are primarily located on DA neurons and act as autoreceptors that inhibit DA activity [50]. Blocking VTA DA autoreceptors with a D2 antagonist disinhibits DA neurons and enhances mesocortical and mesolimbic neuron activity [50]. On the other hand, D2 receptors in the NAcc are located both presynaptically as autoreceptors and postsynaptically on GABAergic medium spiny neurons [51,52]. Interestingly, D2 antagonist infusion into the VTA, but not the NAcc, blocked drug-seeking behavior during the reinstatement test. These data suggest it is the activity of the mesocortical, not the mesolimbic, pathway that is necessary for reinstatement to occur. Previously, cocaine-primed reinstatement of self-administration has been shown to depend on activity of an intact limbic-cortical-striatal pathway [53]. There are reciprocal connections between the VTA and PFC with VTA DA neurons projecting to glutamate neurons in the PFC and glutamatergic neurons projecting from PFC to NAcc [54]. Enhanced VTA DA neuron activity modulates PFC glutamate projection neurons. Specifically, DA increases the amount of time PFC neurons are in their active or “upstate” and suggests DA activates PFC glutamatergic neurons [55]. Sun and colleagues (2005) suggested that increases in PFC DA and NAcc glutamate were both necessary for cocaine-prime reinstatement to occur [56]. Therefore, in the present study, altering VTA mesocortical DA neuron activity with D2 antagonists may have enhanced PFC DA levels, modulated NAcc glutamate levels and resulted in a blockade of cocaine primed reinstatement. Inactivating VTA DA neurons blocked cocaine-seeking behaviors [57]. Given that D2 antagonists in the NAcc did not block cocaine-primed reinstatement suggests that NAcc D2 receptors are not necessary for cocaine-primed reinstatement to occur and that is the specific location of the D2 receptors in the VTA that modulates the expression of drug-seeking behaviors. 

Adolescent brains undergo a physiological shift in primary brain activity from the limbic system during adolescence to more involvement of cortical areas in adulthood [58]. Brenhouse and colleagues [20] reported the density of glutamatergic projection cells from the PFC to the NAcc core progressively increased across age with adults having greater cortical innervations of the NAcc than younger rats. Given the VTA, PFC and NAcc core mediate cocaine-induced reinstatement [21,22,23,24,25,26] and it is these same brain regions that undergo developmental transitions during adolescence [20], it is likely reorganization of neuronal ensembles in the developing brain attribute to enhanced drug use liability during adulthood. The present experiments characterized the effects of early cocaine exposure on cocaine-induced reinstatement of place preferences later on in adulthood. Interpretations of the present data are limited given that an adult rat comparison group was not included in the research design. The present data suggests effects of cocaine exposure during adolescence are long-term and impact drug-seeking behavior later on in adulthood. Furthermore, drug-seeking behaviors were ameliorated by intra-VTA D2 antagonist infusions. It should be noted that results similar to what was obtained in adolescent rats could be obtained from adult rat comparisons. Without an adult comparison group, concluding that adolescents undergo unique plastic changes following drug exposure as compared to adults may be a bit premature. However, previous research has indicated major changes during adolescence occur in DA receptor/transporter density [6,7,8,9,10] and sensitivity of DA autoreceptors [11]. Due to the fact that major restructuring of dopaminergic receptors occur during adolescence and that repeated cocaine exposure disrupts activity of mesolimbic DA, it is likely that repeated cocaine exposure during development has long-term effects on behavioral outcomes requiring an intact and properly functioning mesolimbic DA pathway. Future studies are needed to compare the long-term effects of early cocaine exposure with adult comparisons and if these behavioral effects are mediated in an age-dependent manner by VTA D2 receptors. 

## 5. Conclusions

Taken together, the present experiments show: (1) cocaine place preferences can be expressed during adolescence; (2) exposure to cocaine during adolescence promotes cocaine-seeking behaviors later in adulthood; and (3) cocaine-induced reinstatement of cocaine place preference is mediated by VTA, but not NAcc, D2 receptors. Investigating the interaction between early cocaine exposure and the developing brain is an important step towards modeling human drug use patterns during adolescence and understanding the long-term development of drug dependency. Understanding behavioral and neurochemical responses to factors that induced drug relapse will aid in the development of more effective treatment strategies.
